# Cesium in Biology, Pancreatic Cancer, and Controversy in High and Low Radiation Exposure Damage—Scientific, Environmental, Geopolitical, and Economic Aspects

**DOI:** 10.3390/ijerph18178934

**Published:** 2021-08-25

**Authors:** Sebastiano Venturi

**Affiliations:** Department of Hygiene and Public Health, 61016 Rimini, Italy; dr.venturi.sebastiano@gmail.com

**Keywords:** pancreatic cancer, cesium, radioactive cesium, nuclear fallout, epidemiology

## Abstract

Radionuclide contamination in terrestrial ecosystems has reached a dangerous level. The major artificial radionuclide present in the environment is cesium-137 (137-Cs). In humans, animals, and plants cesium ion (Cs+) behaves like potassium ion (K+) and it is localized mainly inside the cells. Pancreas and salivary glands secrete Cs in the intestine thus eliminating about 14% of ingested Cs with the feces, the remaining 86% is eliminated by the kidney with the urine. Ingested radiocesium can also cause in humans several cases of pancreatitis with secondary diabetes (type 3c), which are both on the rise in the world. The Author studied the correlation between the geographical map of mortality from pancreatic cancer (PC) and the map of nuclear plant accidents, atomic bomb testing, and radioactive fallout. The worldwide death rate of PC is increasing, but the exact cause is still not known. Published data in medical literature at World, European and Italian levels are reviewed and compared. 137-Cs, with a half-life of about 30 years, is still present in the environment for about 300–600 years. Autoradiographic studies in mice have shown that 137-Cs is concentrated in greater quantity in the pancreas, particularly in exocrine cells, where most malignant PCs originate. Some methods of radiocesium removal and PC prevention are also suggested. But there is still a persistent, and not entirely disinterested, the controversy between damage from high and low exposure to ionizing radiations.

## 1. Introduction

**Figure 1:**
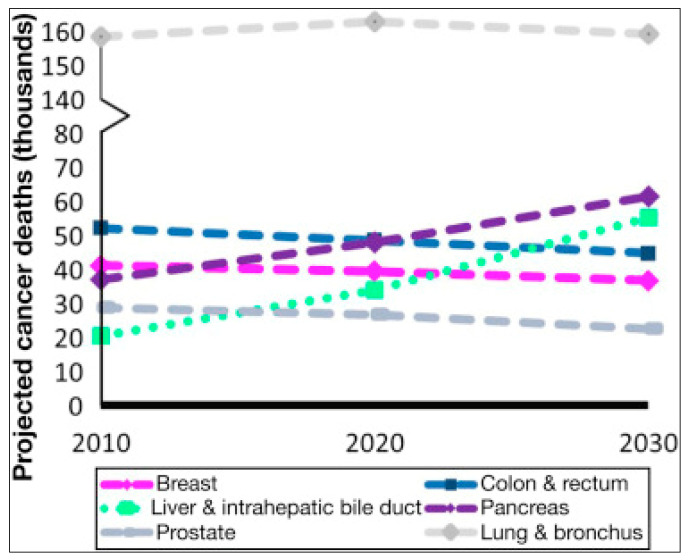
Temporal trends of deaths caused by the most frequent cancers and their forecast in the world from 2010 to 2030.

Radionuclide contamination in terrestrial ecosystems has reached a dangerous level. The major artificial radionuclide present in the environment is radiocesium (137-Cs). Worldwide, both the incidence and death rates of pancreatic cancer (PC) are increasing (Figure 1) [1,2]. 

PC is the seventh leading cause of global cancer deaths in industrialized countries and the third most common cancer in the USA. Based on GLOBOCAN 2018 estimates, PC has been ranked as the 11th most common cancer in the world counting 458,918 new cases and causing 432,242 deaths (4.5% of all deaths caused by cancer) in 2018 [2]. Worldwide incidence and mortality of PC correlate with increasing age and are slightly more common in men than in women. Despite advancement in the knowledge of potential risk factors that cause PC and in newly available tools for early diagnosis, its incidence is estimated to increase and will include 355,317 new cases up to the year 2040 in the U.S. Moreover, the death rate may increase more than that of other cancers because PC, unlike many other cancers, is still incurable. While the exact cause of PC is not known, certain risk factors associated with the disease include tobacco smoking, obesity, and both types of diabetes mellitus [3]. The present paper correlates the increasing PC to the environmental dispersion of radioactive cesium-137.

### 1.1. Controversy on Damage from High and Low Radiation Exposure

#### Scientific, Environmental, Geopolitical, and Economic Aspects

There is a great deal of controversy concerning the accuracy of the methods used to determine the biological damage of nuclear power plant accidents. Since 1986, the total death toll of the disaster has lacked consensus; as the medical journal “The Lancet” and other sources have noted, it remains contested [4]. Determining the elevated risk or a total number of deaths from low doses is completely subjective, and while much higher values would be detectable, lower values are outside the statistically significant reach of empirical science and are expected to remain unknown [5].

Christopher Busby reported [6] that the Ukrainian Ministry of Health, despite its optimistic beginnings went on to warn its readers against consuming local milk, berries, or mushrooms, in a pamphlet stated:

“Dear Comrades! Since the accident at the Chernobyl power plant, there has been a detailed analysis of the radioactivity of the food and territory of your population point. The results show that living and working in your village will cause no harm to adults or children.” 

On 2 March 2012, Katherine Harmon in “Health—*Scientific American*” [7] wrote this article-title:

“Japan’s Post-Fukushima Earthquake Health Woes Go beyond Radiation Effects. Heart disease and depression are likely to claim more lives than radiation after the earthquake, tsunami, and nuclear accident, experts say. Some public health experts agree that the radiation fears were overblown, and comparing with the effects of the radiation exposure from Fukushima the number of expected fatalities are never going to be that large and in terms of the health impact, the radiation is negligible, the radiation will cause very few, close to no deaths.” 

Professor Christopher Busby, Scientific Secretary of the European Committee on Radiation Risk (ECRR) wrote in his book “*Wings of Death: Nuclear Pollution and Human Health*” (1995), that there were many misleading reports that, with apparently good intentions, seriously underestimated the far-reaching consequences of the Chernobyl nuclear catastrophe in 1986. After 1991, also many international diplomats and scientists allied to the nuclear industry evaded or denied the fact of a wide-scale public health disaster caused by radiation exposure. Efforts to spin the story about Chernobyl were largely successful; the official death toll ranges between 31 and 54 people. Busby criticized other researchers studying the health effects of low-dose radiation, and also a report of the United Nations, where was stated that that only 28 people have died since 2005 from radiation emissions in Chernobyl and that there is an “*overreaction*” to radiation low dose. In reality, according to his book, radiation exposure from the disaster caused probably between 35,000 and 150,000 deaths, or more, in Ukraine alone [8].

Drawing on a decade of archival research and on-the-ground interviews in Ukraine, Russia, and Belarus, Kate Brown, professor of Science, Technology, and Society at Massachusetts Institute of Technology unveils the full breadth of the devastation and the whitewash that followed, in her book “*Manual for Survival: A Chernobyl Guide to the Future*” (2019). No major international study tallied the damage, leaving Japanese leaders to repeat many of the same mistakes after the Fukushima nuclear disaster in 2011 [9]. 

Recently (2020), Hauptmann and other 15 international researchers of 8 nations and from Institutes of Biostatistics, Registry Research, Centers of Cancer Epidemiology, Radiation Epidemiology, U.S. National Cancer Institute (NCI), International Agency for Research on Cancer (IARC), and Radiation Effects Research Foundation of Hiroshima, conducted a study. They investigated definitively through meta-analysis the damage resulting from the “low doses”, that have affected the survivor populations of the atomic bomb explosions in Hiroshima and Nagasaki, and also in numerous accidents of nuclear plants that have occurred in the world. These scientists reported in JNCI Monographs*: Epidemiological Studies of Low Dose Ionizing Radiation and Cancer Risk,* that the new epidemiological studies directly support excess cancer risks from low-dose ionizing radiation [10]. 

### 1.2. Table of Radionuclides 

**Figure 2:**
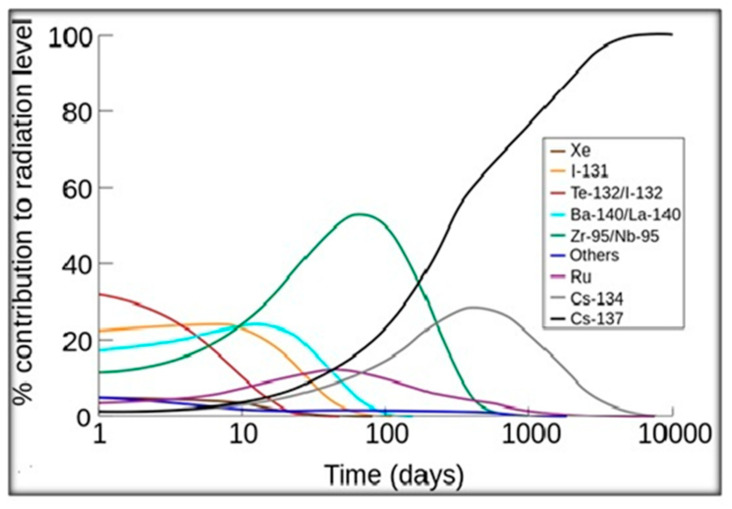
The relative contributions of the major nuclides to the radioactive contamination of the air after an accident. Retrieved on 2009 from [12].

The portion of the total radiation dose (in the air) contributed by each isotope has been plotted against time after the Chernobyl disaster (Figure 2). 137-Cs became the primary source of radiation about 200 days after the accident [11]. Nuclear fission products are the atomic fragments (radionuclides) left after a large atomic nucleus undergoes nuclear fission. Typically, a large nucleus like that of uranium fissions by splitting into two smaller nuclei, along with a few neutrons, the release of heat energy (kinetic energy of the nuclei), and gamma rays. 

### 1.3. The Most Studied Radionuclides: Iodine-131, Cesium-137 

The metabolism of radionuclides, such as cesium and iodine in biology, has been studied by many scientists, initially only to investigate the new radiation damage of atomic explosions after 1945. Only later researchers have investigated the role of these elements in biology and physiology. Among these researchers are Nelson et al. (1961) [13], Ullberg et al. (1961) [14], Pellerin et al. (1961) [15], Rosoff et al. (1963) [16], Lestaevel et al. (2010) [17], and recently Venturi (1999, 2011, 2020) [18,19,20].

But how can we study the low radiation damage of these nucleotides? Certainly not with a direct statistical study of 1 to 1. Since we are dealing also with low radiation doses (much lower than the known published, rapidly lethal doses), which, however, are very prolonged, even for years, the carcinogenic damage is detectable only with a stochastic analysis (on large numbers) because there is only an indirect cause-effect ratio of 1:100, or even more! On the other hand, the oncogenic damage of DNA on one chromosome allele becomes evident only subsequently and may occur after 8–10 or more years, transforming the affected cell into a cancer cell. 

Two different ways of bioaccumulation of radionuclides in the body are:

(1) high but single dose (e.g., 1000 Bq) bioaccumulation, or short half-life radionuclide (such as iodine-131, which has a half-life of 8 days),
(2) low but continuous dose (e.g., 10 or 1 Bq/day), or long half-life radionuclide (such as cesium-137, with half-life = 30.2 years, or cesium-134, with half-life = 2 years).

Iodine-131 may give a higher initial dose, but its short half-life of 8 days ensures that it will soon be gone. Iodine-131 remains in the environment for about only 100 days [19,21]. Cesium-137 decay by gamma and beta (electron) emissions produces highly ionizing radiation [22].

**Figure 3:**
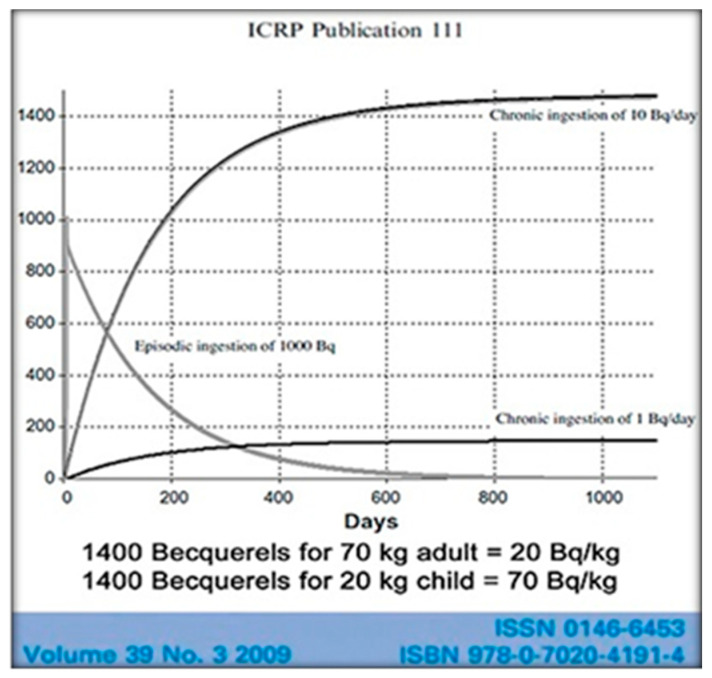
The cumulative biological internal dose (and relative damage) is well represented by the attached graph, where the child is more affected and damaged than the adult [23].

The International Commission on Radiological Protection (ICRP) sets radiation safety standards and recognizes that radiocesium bioaccumulates harmfully in humans. The reported ICRP-figure (Figure 3) compares single ingestion of 1000 Becquerels (Bq) of 137-Cs, a one-time exposure, with the daily ingestion of 10 Bq. On the single exposure, it is noticed that half of the 137-Cs is gone from the body in 110 days. That’s considered to be the biological half-life. Note also that with the routine daily ingestion of 10 Bq of 137-Cs the total radioactivity within the body continues to rise until after about 500 days there are more than 1400 Bq of radioactivity measured in the body [23]. 

## 2. Iodine

Absorption of radioiodine (131-I) can lead to acute and chronic effects. Acute effects from high doses include thyroiditis, while chronic and delayed effects include hypothyroidism, thyroid nodules, and thyroid cancer (TC). It has been shown that 131-I released from Chernobyl caused an increase in the incidence of TC in the former Soviet Union and also in Fukushima, but TC mortality rates are generally low, even at higher tumor stages [19,24]. 131-I may give a higher initial dose, but its short half-life of 8 days ensures that it will soon be gone. 131-I remains in the environment for about 100 days. One measure, which protects against this risk, is taking a dose of potassium iodide (KI) before exposure to radioiodine. The non-radioactive iodide saturates the thyroid, causing less storage in the body, reducing its damage by 99%.

## 3. Cesium

Stable (non-radioactive) cesium (133-Cs) is an alkali metal, water-soluble, which exists naturally at very low concentrations in the soil as the Cs+ ion [22]. Cs has no known beneficial function in animals and plants; however, at high concentrations, it can cause toxicity manifested as growth inhibition.

### 3.1. Radioactive Cesium

Radioactive Cs (137-Cs and 134-Cs), a byproduct of nuclear fission of uranium, are produced from anthropogenic sources and rapidly incorporated into the food chain. Cs is absorbed by plants competitively with potassium and in its radioactive form it is the most dangerous radioisotope to the environment because of its long-term effects. Cesium-137 (137-Cs) half-live is about 30 years, which makes it present in the environment for about 300–600 years. 137-Cs decay by gamma and beta (electron) emissions produces highly ionizing radiation. Beta emission is very dangerous when radioactive Cs is ingested because it deposits all energy in a very short distance of 3–4 mm (several hundred cells in pancreatic tissue!) [22]. 

Flowering plants can transfer radiocesium from soils to honey bees, who can then concentrate the contaminant in honey [25]. In the wake of World War II, the United States, the former Soviet Union, and other countries detonated hundreds of nuclear warheads in aboveground atomic bomb testing. The bombs ejected radiocesium into the upper atmosphere, and winds dispersed it around the world before it fell out. The spread wasn’t uniform, however. For example, far more fallout dusted the U.S. Eastern states, whose populations are the most affected by PC [3].

It has been found that 137-Cs can trace the transport of other radionuclides that have a high affinity for binding to soil particles (silts and clays). In recent years, considerable interest has been applied to the fate and transport of radionuclides, especially after Chernobyl (1986) and Fukushima Daiichi (2011) Nuclear Power Plant disasters. These accidents led to the release of large amounts of radionuclides into terrestrial and aquatic ecosystems [26]. Ingestion of radiocesium can lead to severe acute pancreatitis (and also to an excess of secondary diabetes of pancreatic origin (type 3c), in contaminated population) as reported after a few months in Fukushima prefecture by “Japan Intractable Diseases Information Center, 2014” [27]. The 137-Cs accumulation has also occurred in the Baltic Sea, which is susceptible to pollution as a result of its limited water exchange, shallowness, and large catchment area, and nowadays also from exhausted nuclear deposits of atomic submarines and ships [20]. 

### 3.2. Cesium Metabolism 

In 2020, Venturi reported in the Russian journal “Biosfera” [20] that, contrary to the opinion of many researchers [17] and partly differently from the recent report on the organs of Japanese monkeys [28], 137-Cs is not uniformly distributed in human tissues. For this reason, the radiation damage is greater in Cs-concentrating organs, such as the exocrine pancreas, salivary glands, and intestines. Ingested 137-Cs also largely concentrates on the skeletal muscles. About 86% of adsorbed Cs is excreted in the urine by the kidney, and the remaining 14% is eliminated in the feces, by saliva and pancreatic juice. In humans, the biological half-life of absorbed Cs varies from 50 to 150 days. 

“Prussian Blue” (ferric ferrocyanide) is able to chelate Cs in the intestine and, preventing its reuptake, eliminates it with feces. Nielsen et al. [29] studied the effects of two Prussian blue derivatives on intestinal absorption of 134-Cs in two male volunteers. Their results indicated that administration of Prussian blue (0.5 g) simultaneously with the test meal decreased 134-Cs uptake to about 50%, and that daily administration of 0.5 g × 3 decreased the elimination half time of previously absorbed 134-Cs from about 100 to about 50 days. Melo et al. [30] investigated the effect of age on the decontamination effect of Prussian blue using male beagle dogs injected with 137-Cs. The studied dogs were either immature (about 5 months), young adults (about 2.5 years), or aged (13.5 years). Altagracia-Martínez et al. [31] observed that 137-Cs excretion rates decreased with increasing age. Thus, the reductions in whole-body levels from Prussian blue chelation were 51% in immature, 31% in young adults, and 38% in aged dogs. Prussian blue changed the ratio of fecal to urinary-Cs excretion from 0.8 in untreated dogs, to 2.2 in treated animals. The hepatic 137-Cs levels were reduced in all chelated dogs. 

**Figure 4:**
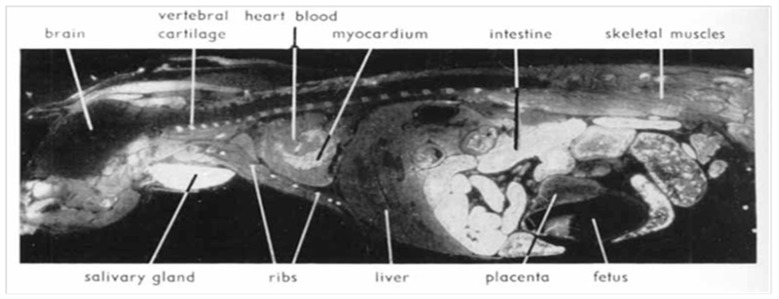
Autoradiograms showing the distribution of 137-Cs in a pregnant mouse 5 min after intravenous injection. White areas correspond to high radioactivity. High uptake in cartilaginous parts of ribs and vertebrae and in salivary gland and intestine. The pancreas shows the same high level of activity as the intestinal mucosa. (Reproduced from Nelson et al. [13] with permission of Acta Radiologica, 1961).

**Figure 5:**
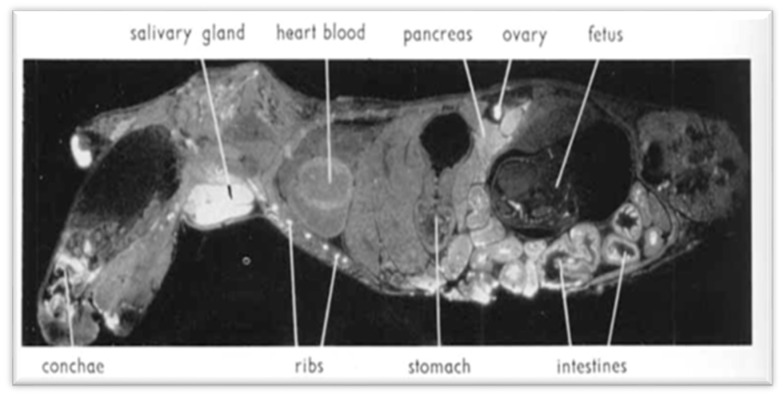
Autoradiogram showing 137-Cs distribution in a pregnant mouse 6 h after intravenous injection. White areas correspond to high radioactivity. Uptake is high in the salivary gland, pancreas, and intestine. In fetuses, concentration is significantly lower than in the mother. In the pancreas, the islets of Langerhans appear to have a slightly lower activity than the acinar tissue. (Reproduced from Nelson et al. [13] with permission of Acta Radiologica, 1961).

Upon renal insufficiency in man and rat, cesium level increases by 100% in the pancreatic tissue [32]. Nelson et al. [13] showed that 137-Cs is concentrated in larger quantity in pancreatic exocrine cells, where most PCs (about 90%) originate, and showed a non-homogeneous selectivity for Cs in different organs: lower in the liver and fetuses (Figure 4 and Figure 5). This was also reported in the autopsy of the liver by Bandazhevsky [33]. 

The high concentration of 137-Cs in the germinal cells of the ovary and the testicle explains the genetic and reproductive damage, sterility, azoospermia, and furthermore, children’s heart diseases and malformations in the population, and also genetic mutations in animals and plants after the Chernobyl accident [13,33]. In the Gomel region, which has been heavily contaminated by the radioactive fallout of the Chernobyl disaster, the damage has also been studied in the rural population since 1990. Children have shown absorption of radiocesium and other radionuclides more than double that of adults. In autopsies of contaminated children, Bandazhevsky found a high accumulation of 137-Cs in the pancreas (and also in thyroid and adrenal glands) detecting levels up to 40–45 times higher than in the liver [33].

### 3.3. Cesium Bioaccumulation and Transfer in Food Chain

Bioaccumulation is the process through which chemicals or radionuclides accumulate and are stored more easily in living organisms than in the environment. The accumulated concentration of chemicals or radionuclides increases then more rapidly than their removal by excretion and metabolism. It is greater in carnivores than in herbivores [26].

### 3.4. Procedures for Radiocesium Decontamination

The removal of the top few centimeters (5–10 cm) of soil and its burial in a shallow trench will reduce human and animal ingestion. Fertilizers containing potassium can be used to dilute radiocesium and limit its uptake by plants. In livestock farming, another countermeasure against 137-Cs is to feed to animals ‘Prussian Blue’, which prevents the chelated radiocesium from being recycled. It is useful also for the treatment of humans [26]. 

## 4. Pancreas

The pancreas is metabolically a very active organ, which despite weighing only 80–100 g in humans it produces from 500 to 2000 mL of pancreatic juice daily. The pancreas has high blood perfusion from the pancreatic arteries and can accumulate inside its cells a considerable amount of radioactive 137-Cs able to damage cell DNA. According to the “Japan Intractable Diseases Information Center”, cases of severe acute pancreatitis increased rapidly by 34% in 2011, about 10 months after the Fukushima accident [27]. Also, diabetes mellitus (of pancreatic origin) increased in the contaminated population of Fukushima. After the Chernobyl disaster, the incidence of children and adolescents with diabetes mellitus (type 1) increased by +25%. Prof. Ito (1994) revealed a 2.1-fold increase in prevalence in males and a 2.0-fold increase in females in Japan in mass screening between 1971 and 1992 for diabetes in adult survivors of the Hiroshima bomb [34]. Martinucci et al. (2002) [35] observed an increased risk of type 1-diabetes in Gomel after the accident compared with before it. Later, a more detailed study by Zalutskaya et al. (2004) [36] also found a significant increase in diabetes in children and adolescents compared with a less contaminated area. Chung et al. (2020) [37] confirmed that diabetes incidence increased 6 years after the Fukushima-Daiichi disaster. These authors stated that this increase was continuing and it has not yet reached its peak. Busby, in “Radioactive Times” [9] reported that in 2016, there were 4 deaths from pancreatic cancer in 16 overall cancers of veterans of the atomic submarine of the U.K. among the entire 20,000 veteran population, while the background rate of pancreatic cancer death is about 1 in 50. 

## 5. Pancreatic Cancer 

### Causes 

The exact cause of PC is not known. General risk factors include the following: Age, with nearly 90 percent found among people aged 55 and older; gender, the cancer being somewhat more common in men than women; obesity; types I and II diabetes mellitus; chronic pancreatitis; liver cirrhosis, *Helicobacter pylori* infection, and cigarettes smoking, the latter cause attributable to almost one-third of all PC. Carcinogens found in tobacco products may damage the pancreas, and smoking may add to the risks associated with other conditions, such as long-term inflammation of the pancreas (chronic pancreatitis). About 10 percent of PC is thought to relate to genetic factors and mutations. However, having a risk factor, or even many, does not mean that you will get cancer [2,3]. Some people who get cancer may have few or no known risk factors. Pancreatic ductal adenocarcinoma (PC) has a very poor prognosis. Typically, only 24% of people survive 1 year after diagnosis, and 9% live for 5 years. Only 1–2% of pancreatic tumors are of the neuroendocrine type, being derived from the cells that produce pancreatic hormones. Such tumors are less aggressive compared with adenocarcinomas. 

## 6. Epidemiological Correlation between Territories with High PC and High Radionuclide Contamination

**Figure 6:**
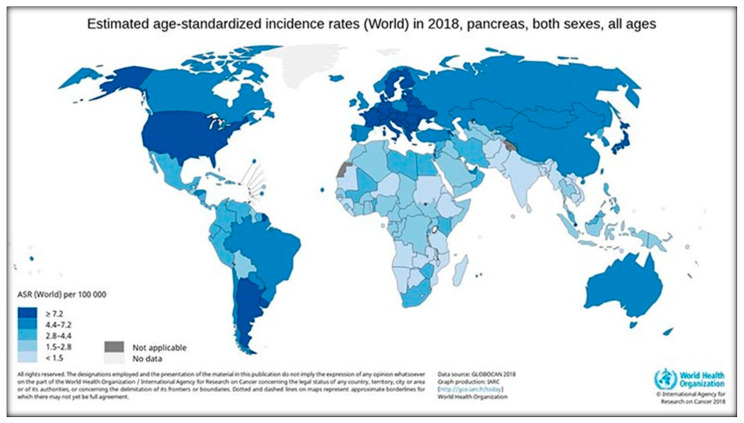
The incidence rates of PC in the world according to IARC [2,3].

The incidence of PC varies across regions and populations (Figure 6)**.** In 2018, 458,918 new cases of PC were registered worldwide, representing 2.5% of all cancers. The age-standardized rate (ASR) incidence was highest in Europe (7.7 per 100,000 people) and North America (7.6 per 100,000 people), followed by Oceania (6.4 per 100,000 people). The lowest rate was observed in Africa with an estimated incidence of 2.2 per 100,000 people [18,27]. Differences in incidence rates were 30-fold between the populations featuring the highest rate (Hungary: 10.8), and the lowest rate (Guinea: 0.35) [3]. The high PC level in Oceania and Australia, where there are no power plants, is probably related to atomic bomb testing.

## 7. PC in Japan

**Figure 7:**
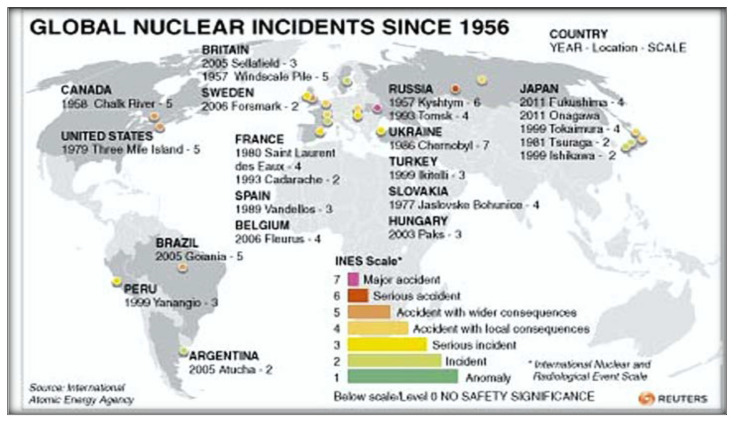
Major nuclear accidents around the world (adapted from [40]).

In Japan, PC is the fourth leading cause of cancer deaths. It is an aggressive disease where approximately 60–80% of patients already have distant metastasis at presentation with a poor survival rate. Especially after the nuclear accident of Fukushima, the PC has an unusually high frequency. The Japanese “Global Data” in 2019 predicted that this cancer will continue to increase in Japan in the next ten years, despite the decrease in the Japanese population. Epidemiologists predict an increase in cases from 42,000 cases in 2019 to 48,000 cases in 2029, with an annual growth rate (AGR) of 1.50%. The incidence of PC in Japan has increased continuously especially in the last decades. The rate of incidence in 2019 was almost double that of the US and about 50% higher than that of France, Germany, Italy, Spain, and the United Kingdom. This trend will likely continue in the next decades. According to Japanese researchers, the cause of this increase is unknown. While smoking, old age and obesity are strong risk factors, the Japanese population is not at greater risk of obesity and smoking-related diseases compared to other nations. Also, regarding the risk factor-age, Japan has an aging population, and it is susceptible to this type of cancer, similar to that of the aforementioned European nations. According to ‘Japanese Global Data Healthcare’, Pharmaceutical Technology (2020) [38], Japan has an unusually high burden of pancreatic cancer. Epidemiologists forecast that this cancer burden will continue to rise in Japan in the next ten years despite the decrease in the Japanese population and they forecast an increase in diagnosed incident cases of PC in Japan from 42,000 cases in 2019 to 48,000 cases in 2029, at an annual growth rate (AGR) of 1.50%. Prior to the 2011 Tōhoku earthquake and tsunami, Japan had generated 30% of its electrical power from nuclear reactors and planned to increase that share to 40%. Nuclear power energy was a national strategic priority in Japan. As of March 2020, of the 54 nuclear reactors in Japan, there were 42 operable reactors but only 9 reactors in 5 power plants were actually operating. A total of 24 reactors are scheduled for decommissioning or are in the process of being decommissioned [39]. The areas where both PC rates and nuclear power production are high often coincide. Higher levels of nuclear power production are prone to higher risks of accidents. Chernobyl was the most serious nuclear accident in the history of nuclear power plants [5,40]. But many other minor accidents have happened in various countries and they weren’t communicated (Figure 7).

### 7.1. 137-Cs vs. PC in Europe

In the early 1970s, mortality from PC was low in Europe, stomach cancer being among the leading causes of cancer death [18]. Nowadays, after about 50 years, the trends in these cancers are reversing. Since the probable PC latency is of about 8–10 years, it is likely that the radioactive fallouts of the numerous atomic bomb tests that were carried out mainly in the years 1960–1970 and from nuclear power plant accidents have had an effect. According to estimations of released radioactivity in nuclear weapons testing fallout and nuclear accidents, the radioactivity of 137-Cs released into the atmosphere from nuclear weapons testing was far greater than that from nuclear accidents:

- ~950 PBq from nuclear weapons testing [41]
- ~85 PBq from Chernobyl [42],
- 12–41 PBq from Fukushima [43].

**Figure 8:**
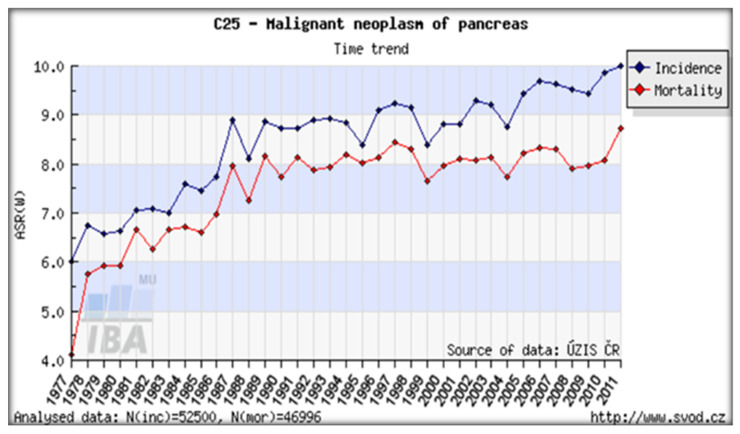
Trends of PC incidence and mortality (1977–2011) in a country of Central-Eastern Europe particularly hit by the Chernobyl radioactive cloud. PC incidence and mortality rates in the Czech Republic (both sexes). Data source: CNCR [47].

**Figure 9:**
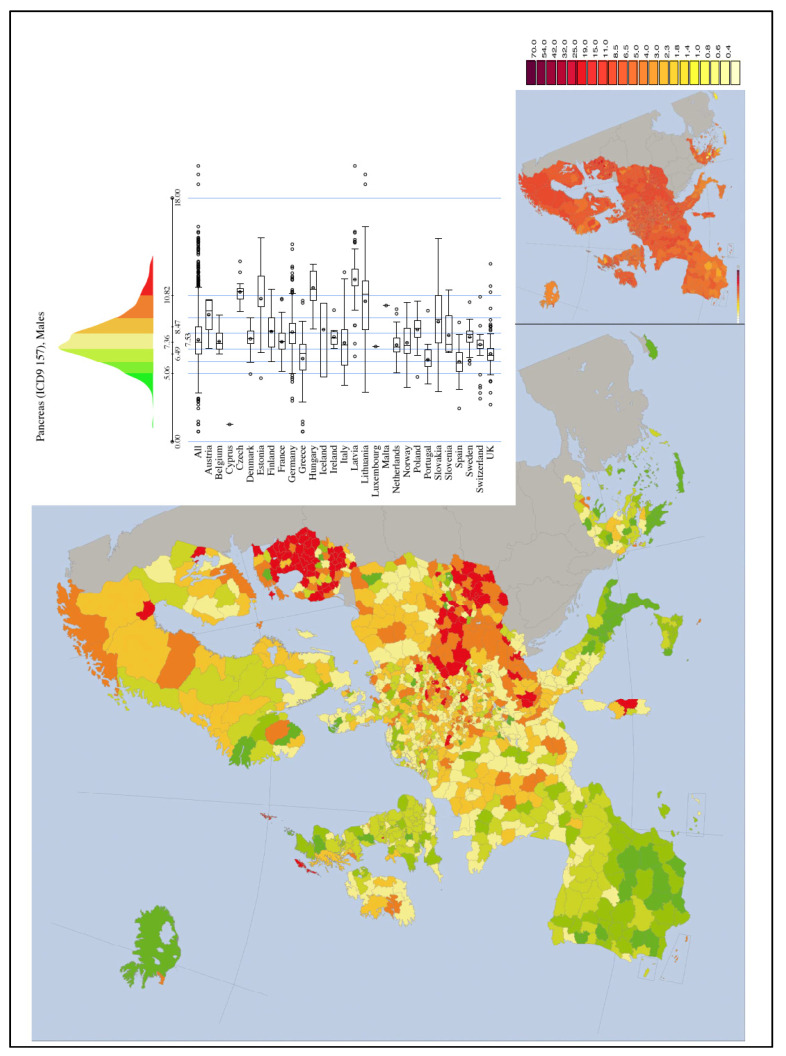
PC mortality (Males) map in Europe from IARC (1993–1997) [50].

Indeed PC increase begins in the early 1980s and, in Europe, after the 1986 Chernobyl accident (Figure 8 and Figure 9).

In the counties of Sweden, Edling et al. (1982) reported that low-dose background radiation exposure significantly correlates with PC (males, r = 0.59; and, females, r = 0.40) [44]. In Ukraine and its neighbor country Belarus, Bandazhevsky et al. [33], Zrielykh et al. [45], and Leung et al. [46] reported an increased incidence of PC and other tumors 20–30 years after Chernobyl.

### 7.2. 137-Cs vs. PC in Italy

In Italy, the incidence of PC is growing significantly (+0.4% annually), with a clear North-to-South gradient: compared to Northern Italy, in central Italy PC incidence levels are 29% lower in males and 26% lower in females, and Southern Italy, they are 25% and 28% lower, respectively. PC ranking among the causes of death from tumors is 4th in females, the total (M + F) PC mortality is 6%. These data correlate with the current exposure to 137-Cs in Italy [48,49]. In Italy, the radioactive cloud of Chernobyl also hit more the Northern part because the cloud came from Northern Europe and covered about half of Italy, while Southern Italy was almost completely untouched.

**Figure 10:**
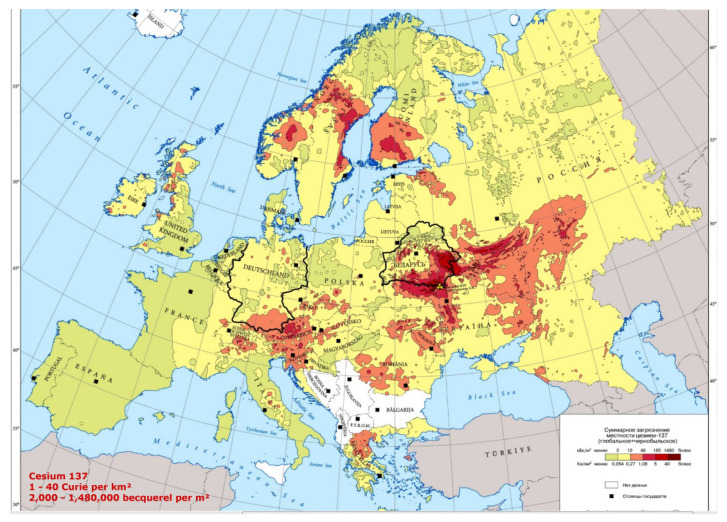
137-Cs distribution of the Chernobyl radioactive cloud in Europe. (Source: Office for Official Publications of the European Communities, Luxembourg, 1998).

In 2016, social scientists and energy policy experts have reported that worldwide there have been 99 accidents at nuclear power plants from 1952 to 2009 (IAEA) [5]. Traces of the radioactive elements released by the 1986 Chernobyl accident are still present at higher levels in Northern Italy, Alsace, Eastern France, and Southern Germany (Figure 10). On the contrary, traces of atomic bomb testing from the 1960s are more evenly distributed, with peaks in central Southern France, in the Massif Central region, in the Ardennes area, and Britain. The highest levels of 137-Cs are found on the surface layers of the soil, from where they are absorbed by plants, fruit, and fungi and enter the food chain, and finally, in the human and predatory animal bodies, where environmental 137-Cs undergoes greater and more harmful bioaccumulation. The Chernobyl accident took place on 26 April 1986; some 10–20 million people were exposed to significant levels of fallout; several hundred workers at the plant received whole-body radiation, 134 developed acute radiation sickness and, of these, 28 died within four months. In Belarus, almost exactly half the cases occurred in the region that is closest to Chernobyl, which received the highest fallout. The world incidence of childhood TC can be assumed as approximately 1/million children/year, but in Belarus as a whole, the incidence by 1995 was almost 30-fold higher, and in Gomel Region, it reached approximately 100/million children/year [3,5,51]. Despite this, TC mortality was and is nowadays low.

**Figure 11:**
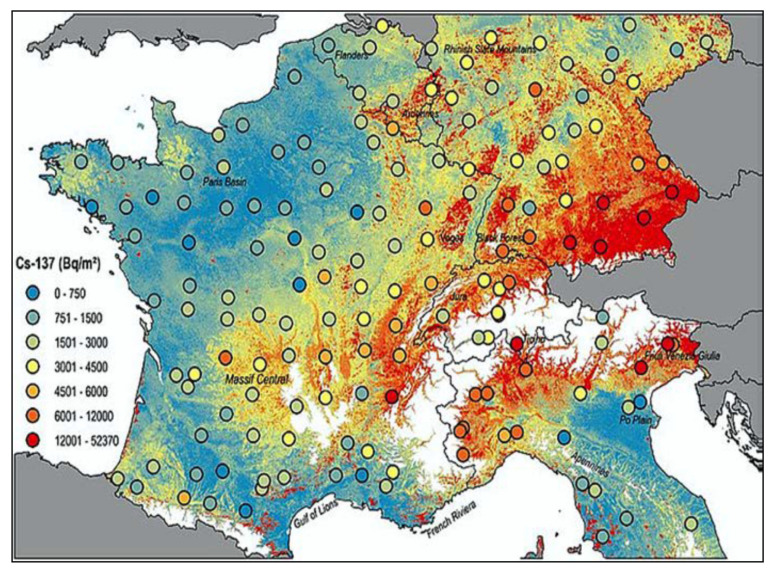
The new map of cesium-137 contamination in Europe in 2020 [52].

Meusburger et al. [52] set out to make a radioactivity inventory on a continental scale, by analyzing the Chernobyl and total 137-Cs fallout across national boundaries, focusing on a selection of soil samples collected in France, Belgium, Southern Germany, Switzerland, and Northern Italy (Figure 11). High 137-Cs is associated with high altitude. In the mountains, rainfalls enhance radioactive fallouts. In the mountainous Northern Italy (Alps) and the hilly Central Italy (Apennines), PC incidence is higher than in Southern Italy and in islands, where PC mortality compared to that in Northern Italy is −29% in males and −26% in females respectively. In 2000, Moseman [53] reported that meat of over 1000 wild boars killed in Germany in the previous year was contaminated with 137-Cs from Chernobyl, with over 600 Bq allowed per kg of meat. It has been also estimated that 137-Cs from the Chernobyl accident is expected to be detectable in Norwegian cattle and sheep, for over 100 years to come [54]. The Norwegian Food Safety Authority ordered the farmers to feed their animals with safer food if radioactivity is too high. Animals can be also fed with the cesium chelator “Prussian Blue”. Recognized sure effects of heavy radionuclide exposure are many, including radiation sickness, cataracts, thyropathologies, blood diseases, leukemia, tumors, heart, and neuropsychological pathologies, etc. 

In 2020, in Ukraine, the number of PC cases increased by 8.7% (7.3% in men and 10.3% in women) in 2013, in comparison with 2003. PC incidence in children (age 0–17 years) was zero in 2003 and 4 in 2013. The age-standardized PC incidence rate was 5.9 in 2003 (8.6 in men; 4.0 in women) and 6.8 in 2013 (9.8 in men; 4.7 in women). The age-standardized mortality rate was 5.0 in 2003 (7.4 in men; 3.4 in women) and 5.5 in 2013 (7.9 in men; 3.9 in women). It should be noted, that in 2013, four children had PC, whereas normally in the rest of the world children are rarely affected [45].

## 8. Conclusions

New metabolic pathways of Cs in the pancreas, salivary glands, and intestine are reported. The stochastic analysis of PC epidemiology may be the key to interpreting the etiology of PC, and some inflammatory pancreatic diseases and related types (3c) of diabetes, which are both on the rise in the world, and thus, the cornerstone of developing a possible effective prevention strategy. The reported data warrant investigations into an association between radioactive Cs and PC. If this correlation will be confirmed, preventive action may be possible by using table salt enriched in potassium and by a diet with fruits and vegetables, rich in potassium. In addition, the soil enriched with potassium is able to reduce 137-Cs uptake by plants [25]. Food must also come from areas not contaminated by radioactivity. In cases of strong accidental ingestion of this radionuclide, the chelating action of the “Prussian blue” has been indicated [55,56].

Fossil oil will likely run out within the next 50–80 years. Alternative energies: sun, wind, hydro, tides, etc. will probably not be able to provide us with the amount of energy needed for a world population, which perhaps will reach 10 billion. All this could cause hunger, poverty, wars, dictatorships, etc. 

What energy can we use and at what risk? 

I hope that this research will help to defend, in part, the world population from the looming risks of nuclear fission.

## Data Availability

Not applicable.
